# Supplementary material for: Mechanism of the electroneutral sodium/proton antiporter PaNhaP from transition-path shooting
Source: Nat Commun. 2019 Apr 15;10:1742. doi: 10.1038/s41467-019-09739-0 (PMC6465308; doi:10.1038/s41467-019-09739-0)
Supplement: Supplementary file 1 — Supplementary Information [file 41467_2019_9739_MOESM1_ESM.pdf]

Supplementary Information for

Mechanism of the electroneutral sodium/proton antiporter PaNhaP from transition-path shooting

K. Okazaki *et al.*

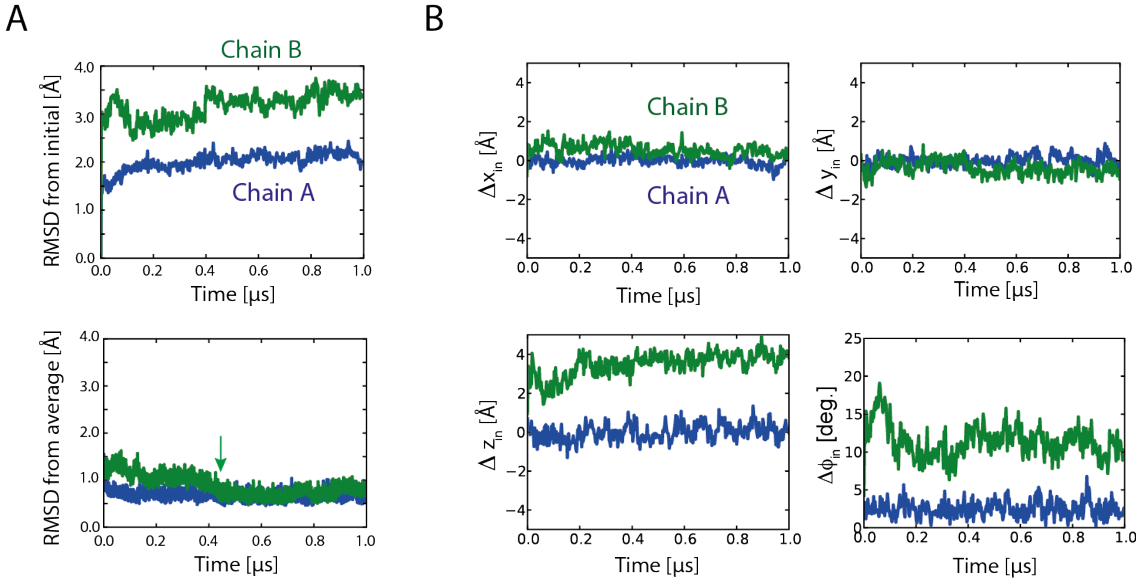

Supplementary Figure 1 | Equilibrium MD simulation of asymmetric dimer following targeted MD simulation. (A) RMSD of backbone atoms (Cα, C, O, N) from (top) the initial and (bottom) the average structures over the last 0.5 μs. The green arrow indicates the relaxation of protomer B towards the final structure at ~0.4 μs, associated with helix-5 motion. (B) Translational and rotational motions of the six-helix-bundle domain relative to the average inward-open structure.

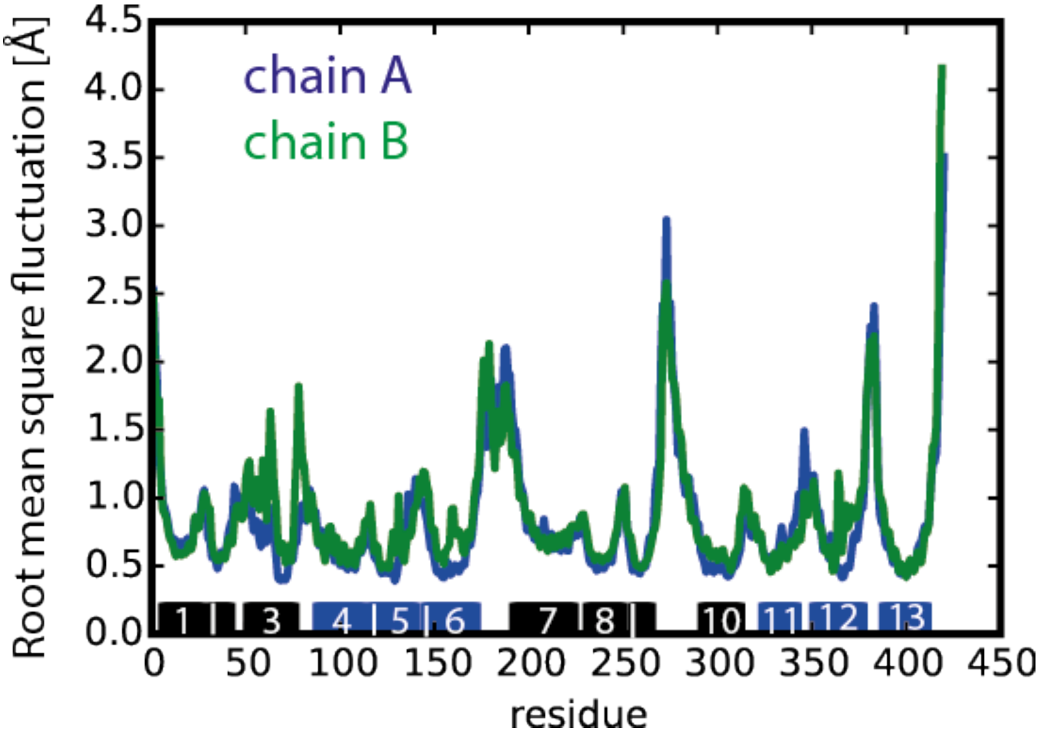

Supplementary Figure 2 | Root-mean-square fluctuations of the Cα backbone in the asymmetric dimer. The fluctuation profiles were obtained as a function of residue number during the second half of the free simulation of the asymmetric dimer. In the 1-μs simulation, the chain-A and chain-B protomers assumed inward- and outward-open conformations, respectively. The bars on the bottom show the helical regions used for structural superposition; numbers in the bars represent the helix number, coloured in blue and black for the six-helix-bundle and dimerization domains, respectively.

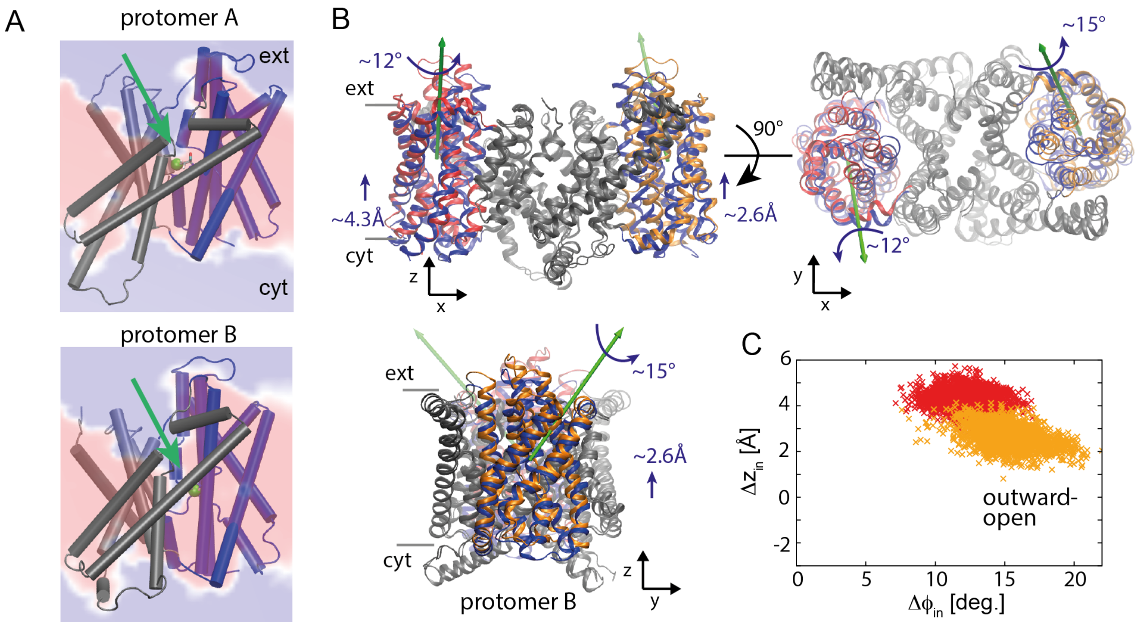

Supplementary Figure 3 | Symmetric outward-open structure from targeted MD and 1-μs free simulation. (A) Outward-open structures of protomers A and B with a section through the average water density in the 1-μs free MD simulation of the symmetric dimer (red: no water; blue: water at bulk density). The green sphere indicates Hδ of the protonated Asp159. (B) Average dimer structure superimposed onto the average inward-open structure in (top) front and (bottom) side views. The green arrows show the axes of rotations of the six-helix-bundle domains. (C) Angle and z-coordinate changes of the six-helix-bundle domains of protomers A and B relative to the average inward-open structures (red and orange crosses, respectively).

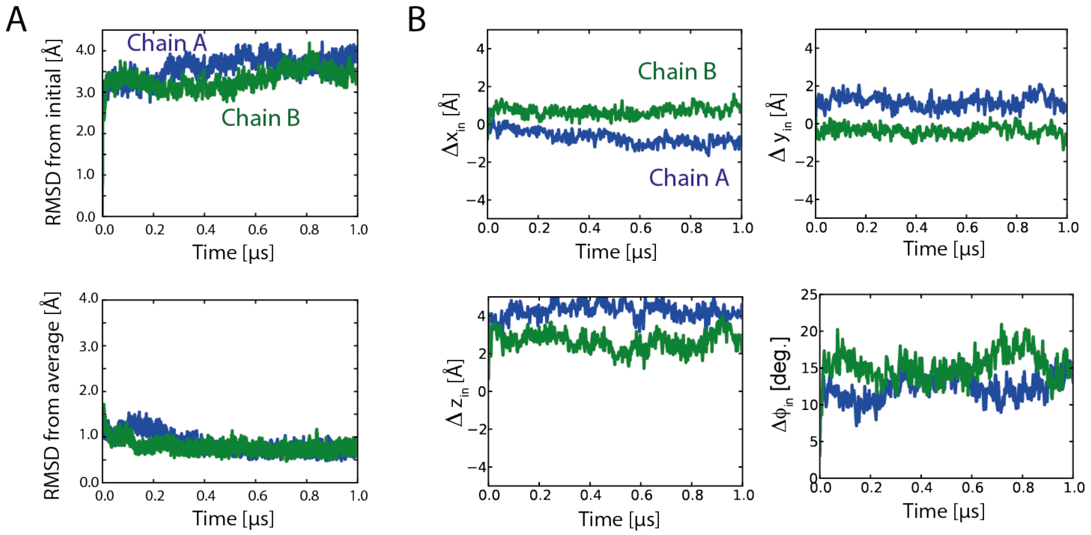

Supplementary Figure 4 | Equilibrium MD simulation of symmetric dimer following targeted MD simulation. (A) RMSD of backbone atoms (Cα, C, O, N) from the initial (top) and average structures (bottom; averaged over the last 0.5 μs). (B) Translational and rotational motions of the six-helix-bundle domain relative to the average inward-open structure.

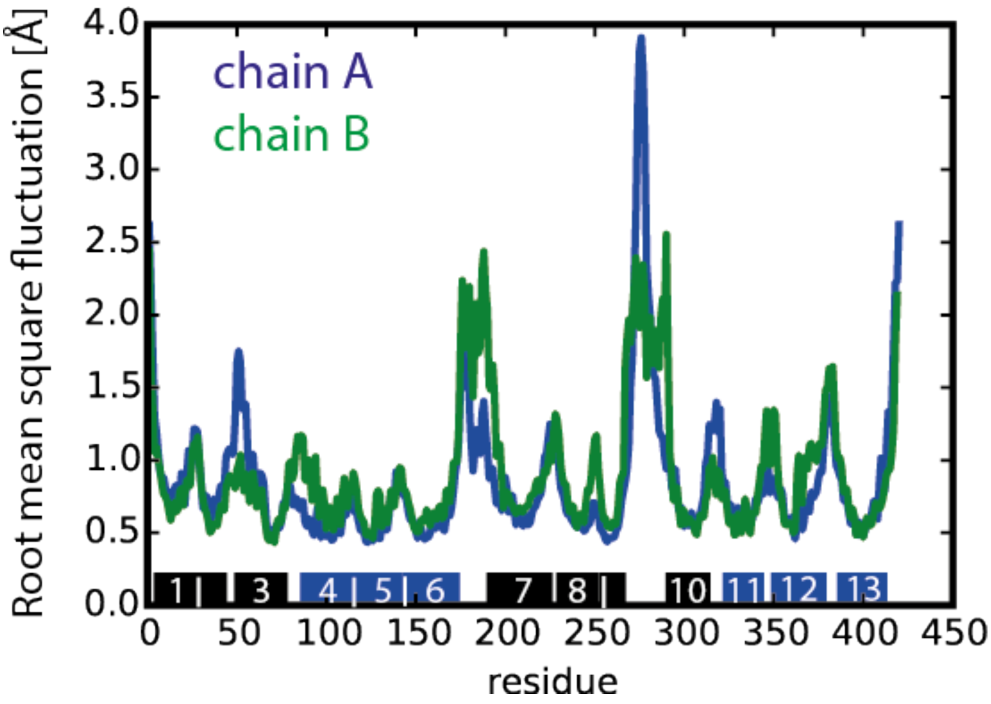

Supplementary Figure 5 | Root-mean-square fluctuations of the Cα backbone in the symmetric dimer. In the 1-µs simulation, both protomers assumed outward-open conformations. The bars on the bottom show the helical regions used for structural superposition; numbers in the bars represent the helix number, coloured in blue and black for the six-helix-bundle and dimerization domains, respectively.

Successful transition paths of H^+, T=310K

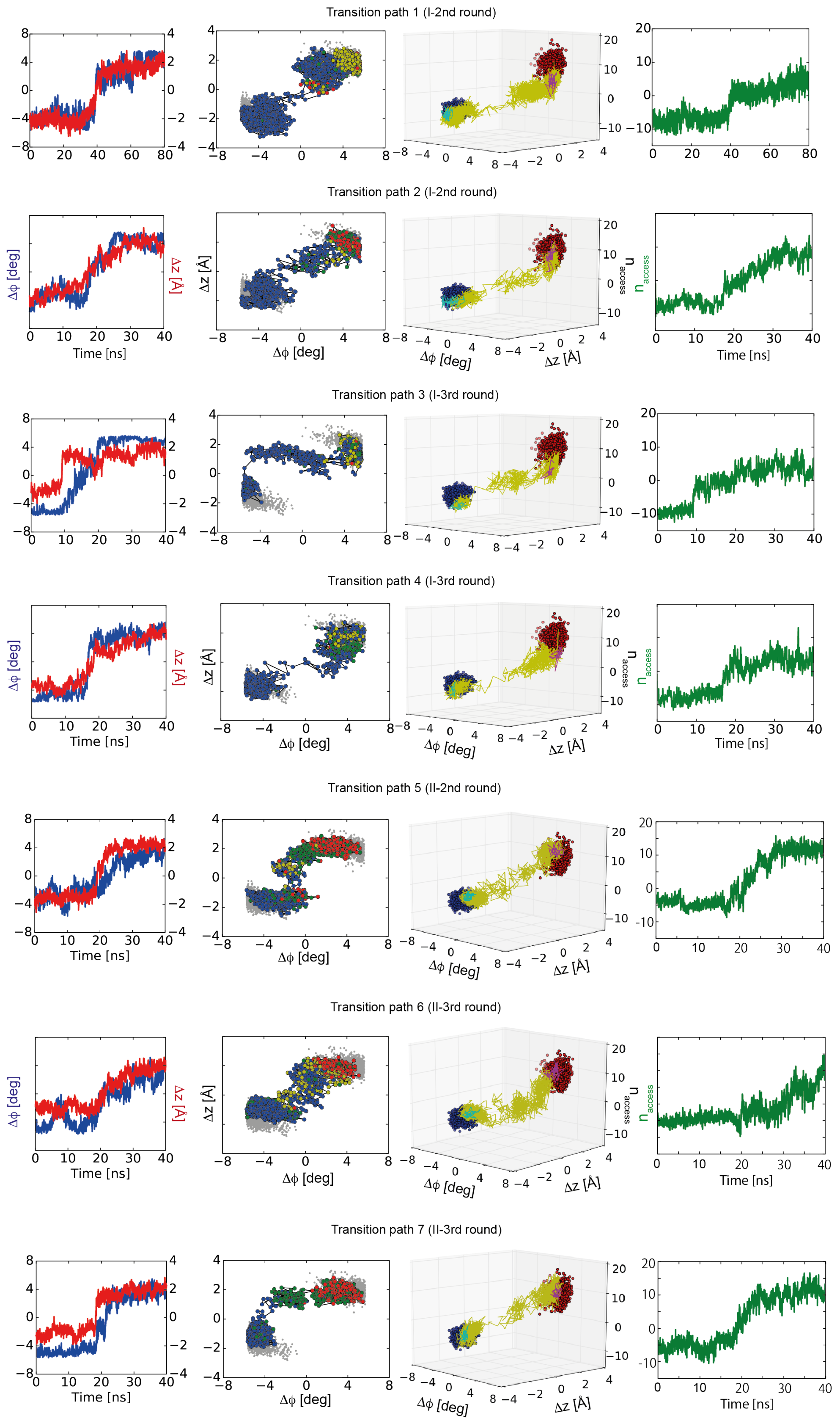

Supplementary Figure 6 | Transition paths with protonated Asp159 at 310 K. Column 1 shows time series of the order parameters (blue/left axis: $\Delta\phi$; red/right axis: Δz). Column 2 shows a projection of equilibrium runs (gray) and transition paths onto the $\Delta\phi$-Δz plane. Inward-open, occluded, connected, and outward-open states are shown as blue, green, yellow, and red dots, respectively. In column 3, the hydration order parameter n_{access} is included as a third coordinate, with the corresponding time series shown in column 4.

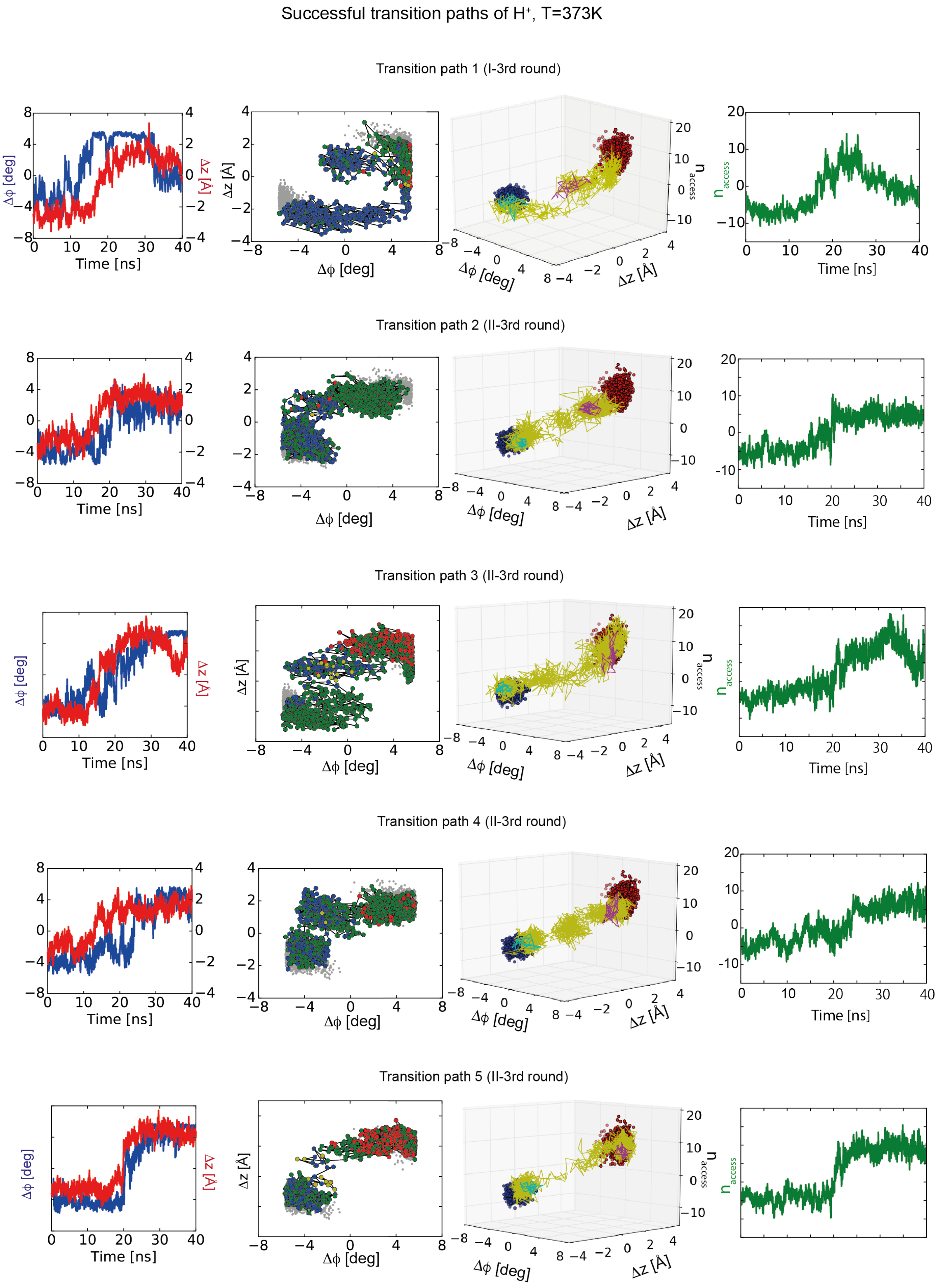

Supplementary Figure 7 | Transition paths with protonated Asp159 at 373 K. Successful transition paths with protonated Asp159 at 373 K. Column 1 shows time series of the order parameters (blue/left axis: Δϕ; red/right axis: Δz). Column 2 shows a

projection of equilibrium runs (gray) and transition paths onto the $\Delta\phi$-Δz plane. Inward-open, occluded, connected, and outward-open states are shown as blue, green, yellow, and red points, respectively. In column 3, the hydration order parameter n_{access} is included as a third coordinate, with the corresponding time series shown in column 4.

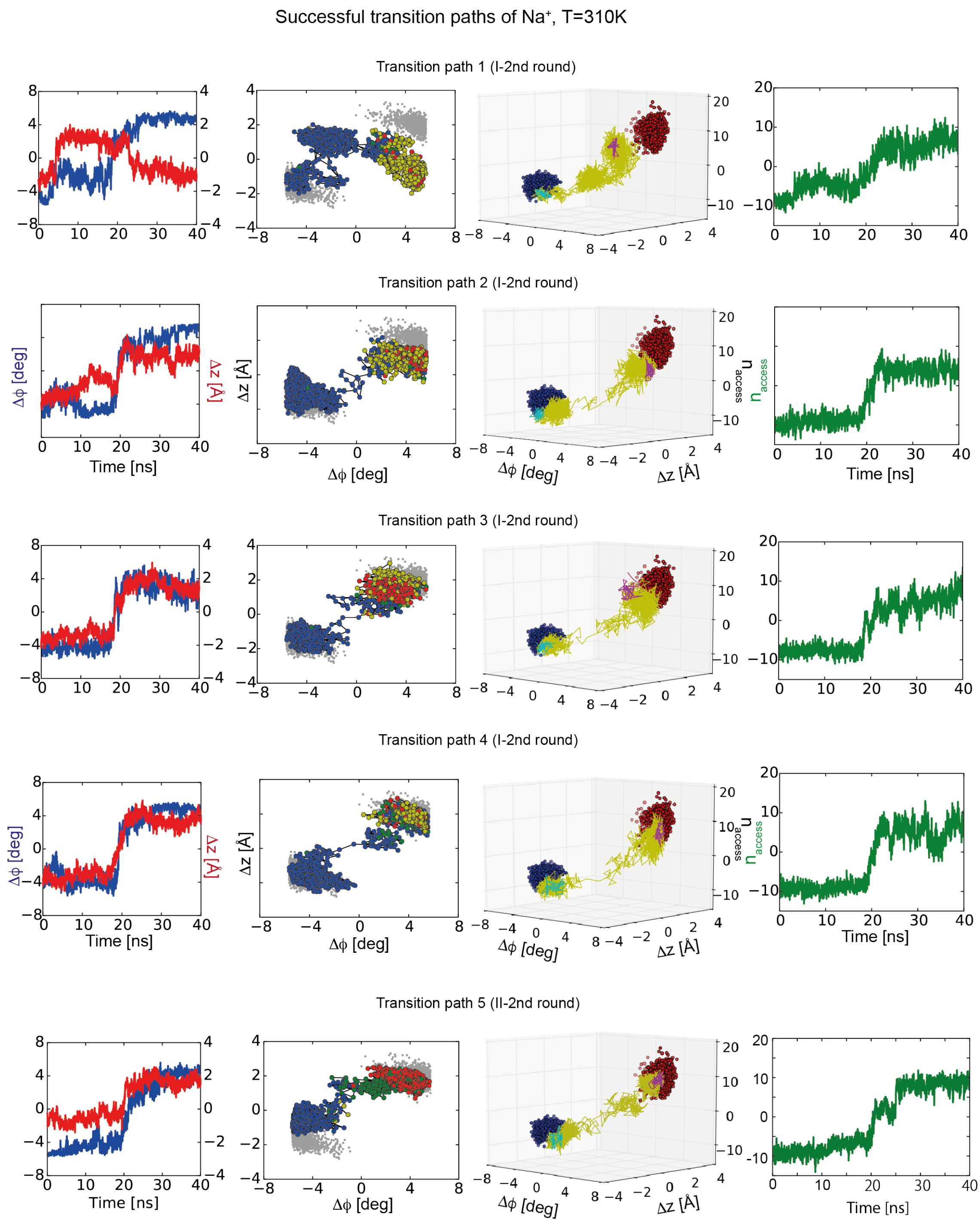

Supplementary Figure 8 | Transition paths with Na^+ bound at 310 K. Column 1 shows time series of the order parameters (blue/left axis: Δϕ; red/right axis: Δ*z*). Column 2 shows a projection of equilibrium runs (gray) and transition paths onto the Δϕ-Δ*z* plane. Inward-open, occluded, connected, and outward-open states are shown as blue, green, yellow,

and red points, respectively. In column 3, the hydration order parameter n_{access} is included as a third coordinate, with the corresponding time series shown in column 4.

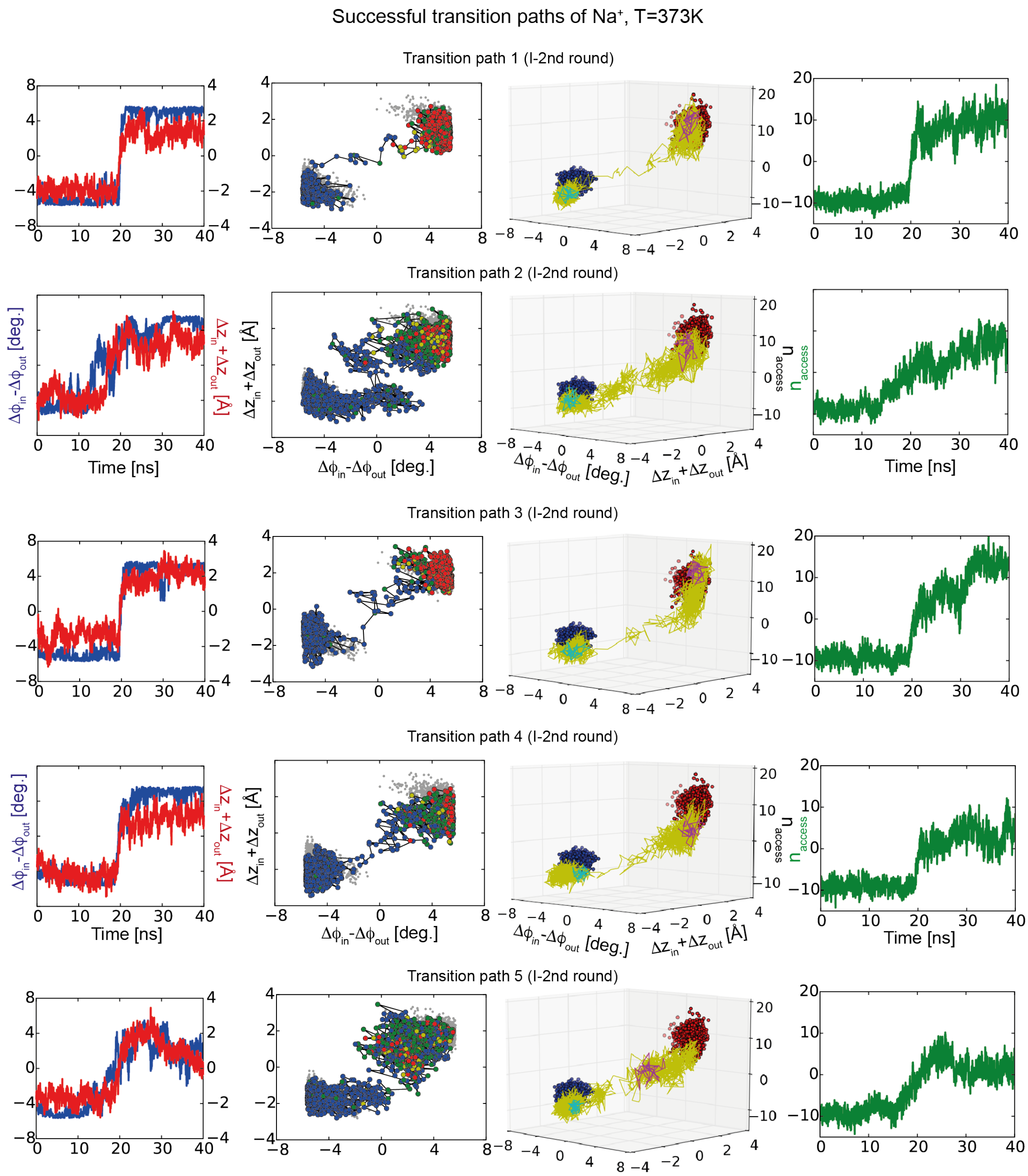

Supplementary Figure 9 | Transition paths with Na^{+} bound at 373 K. Column 1 shows time series of the order parameters (blue/left axis: $\Delta\phi$; red/right axis: Δz). Column 2 shows a projection of equilibrium runs (gray) and transition paths onto the $\Delta\phi$-Δz plane. Inward-open, occluded, connected, and outward-open states are shown as blue, green, yellow, and red points, respectively. In column 3, the hydration order parameter n_{access} is included as a third coordinate, with the corresponding time series shown in column 4.

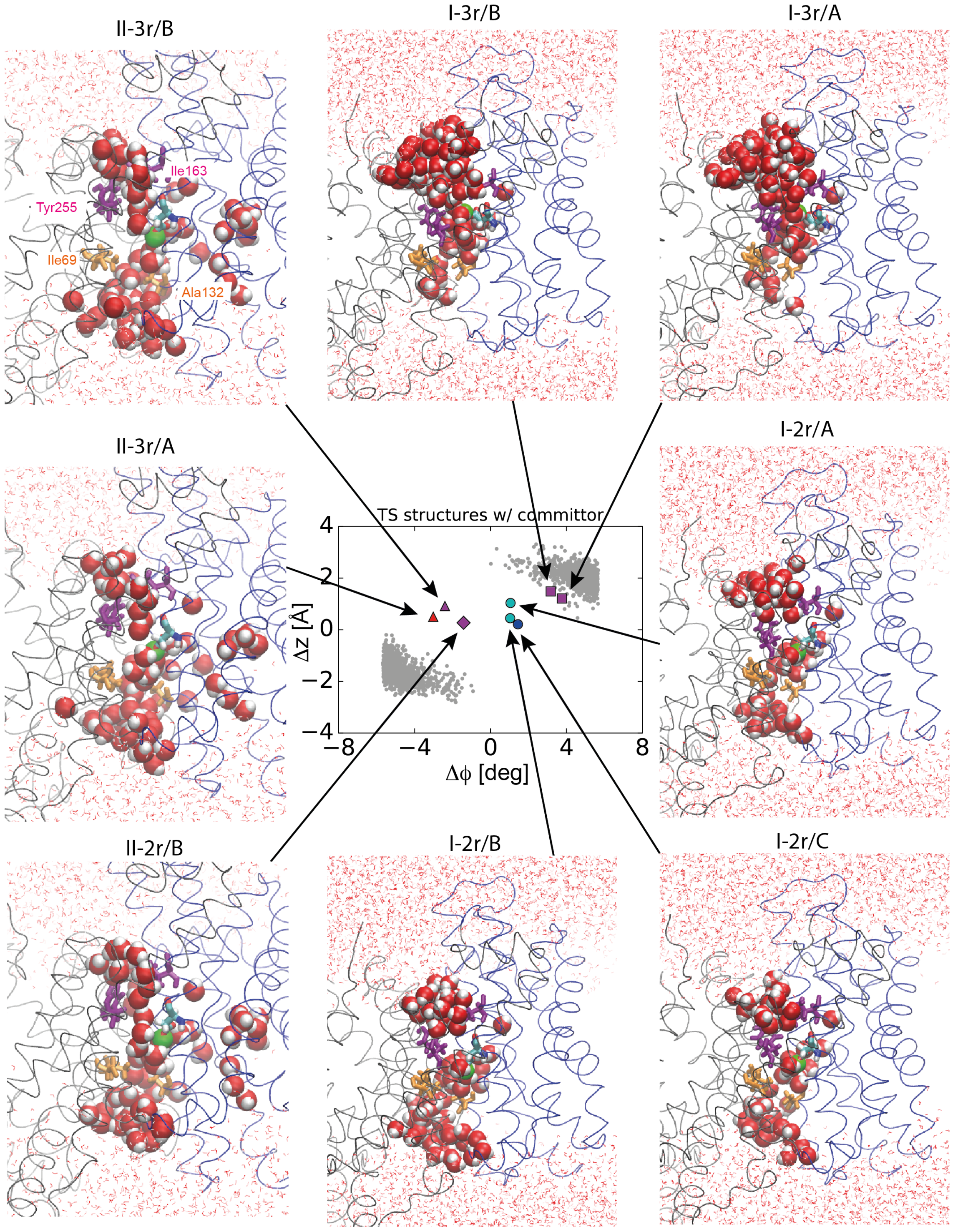

Supplementary Figure 10 | Transition states. Top and bottom rows: Gallery of eight transition states from rounds 2 to 3 of transition-path shooting starting from initial path I and II, respectively. Orange and magenta sticks represent the inward and outward access-controlling residues, respectively. Asp159 is shown in stick representation and the bound H^+ as a green sphere. Water molecules within 15 Å of the bound H^+ of Asp159 are shown

as spheres; other water molecules are shown as lines, and the protein backbone is shown as a tube. Centre: The transition-state structures are mapped onto the $\Delta\phi$-Δz surface. The structures for the second and third rounds of transition-path shooting starting from initial path I are shown as circles and squares, respectively. The structures for the second and third rounds of transition-path shooting following the initial path II are shown as diamonds and triangles, respectively. Estimated committor probabilities to the outward-open state are indicated by colour: >60% (red), 40-60% (magenta), 20-40% (cyan), <20% (blue). The gray dots represent $\Delta\phi$-Δz of structures sampled during last half of the 1 μs equilibrium simulation of the asymmetric dimer (protomer A: bottom left; protomer B: top right).

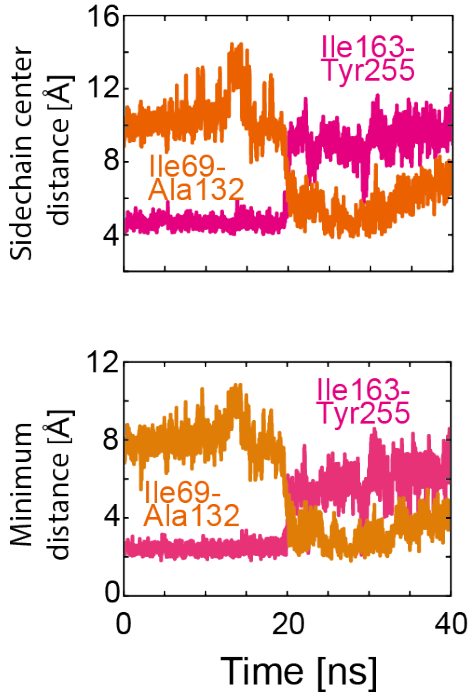

Supplementary Figure 11 | Gate residue distances. (Top) Sidechain-center distances and (Bottom) minimum distances of the gate residues are shown in magenta. Shown in orange are distances of residues reporting on the solvent accessibility of the ion binding site from the cytosolic side.

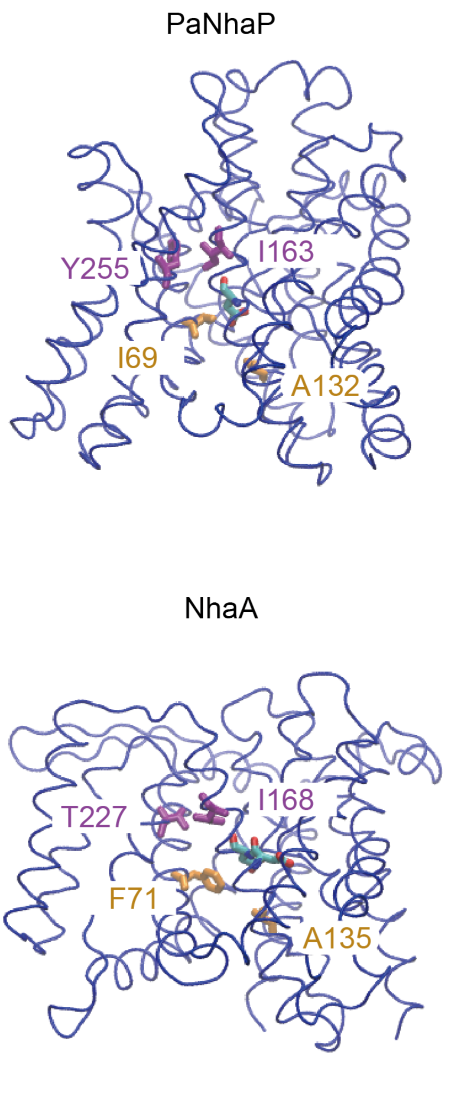

Supplementary Figure 12 | Predicted gate residues from structural alignment. Hydrophobic gate residues are shown in magenta. Also shown in orange are residues involved in controlling solvent access on the cytosolic side. Conserved aspartates are coloured by atom.

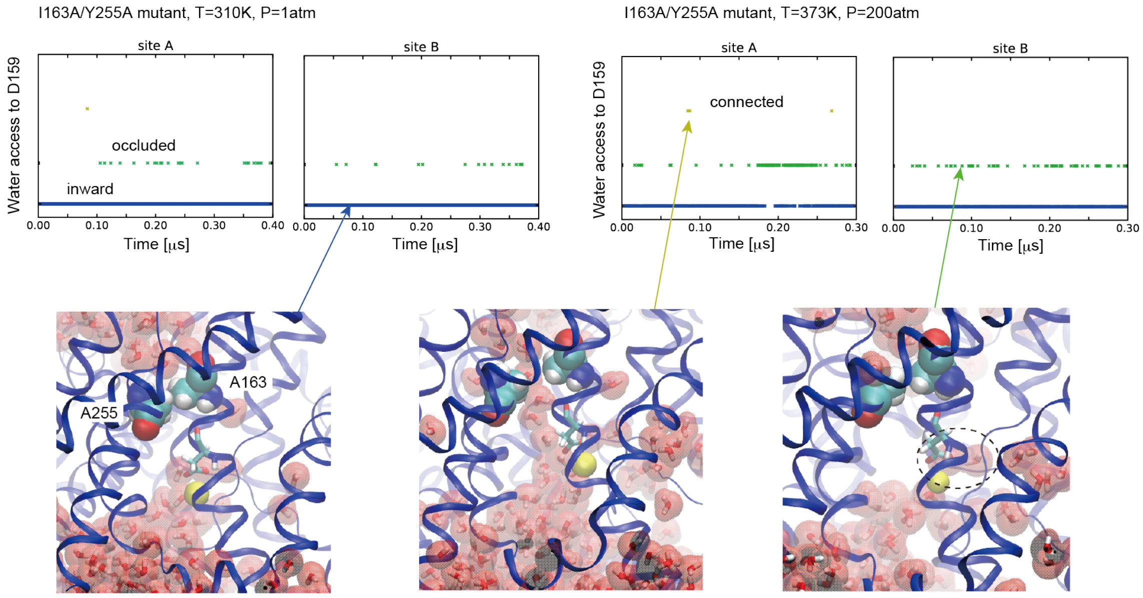

Supplementary Figure 13 | Effect of mutations in hydrophobic gate on solvent access in MD simulations. Water access state of the gate mutant I163A/Y255A in the inward-open conformation from equilibrium MD is shown at T=310 and 373 K with bound Na^{+}.

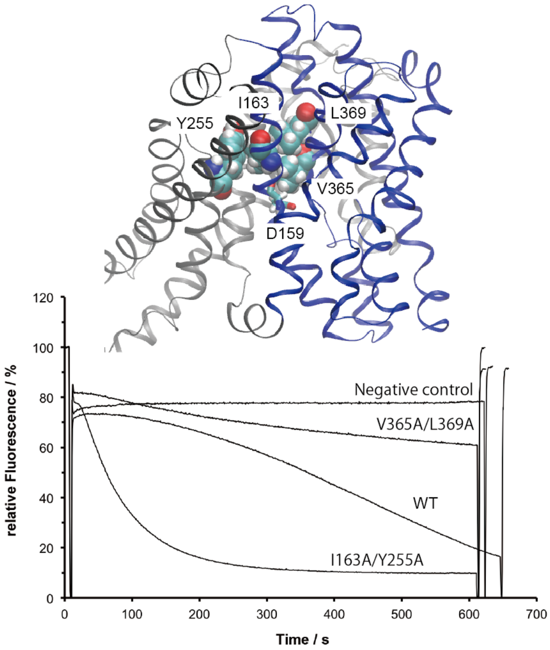

Supplementary Figure 14 | Mutations of the hydrophobic gate. Mutated residues on the structure and fluorescent measurements of transport activity of the mutants.

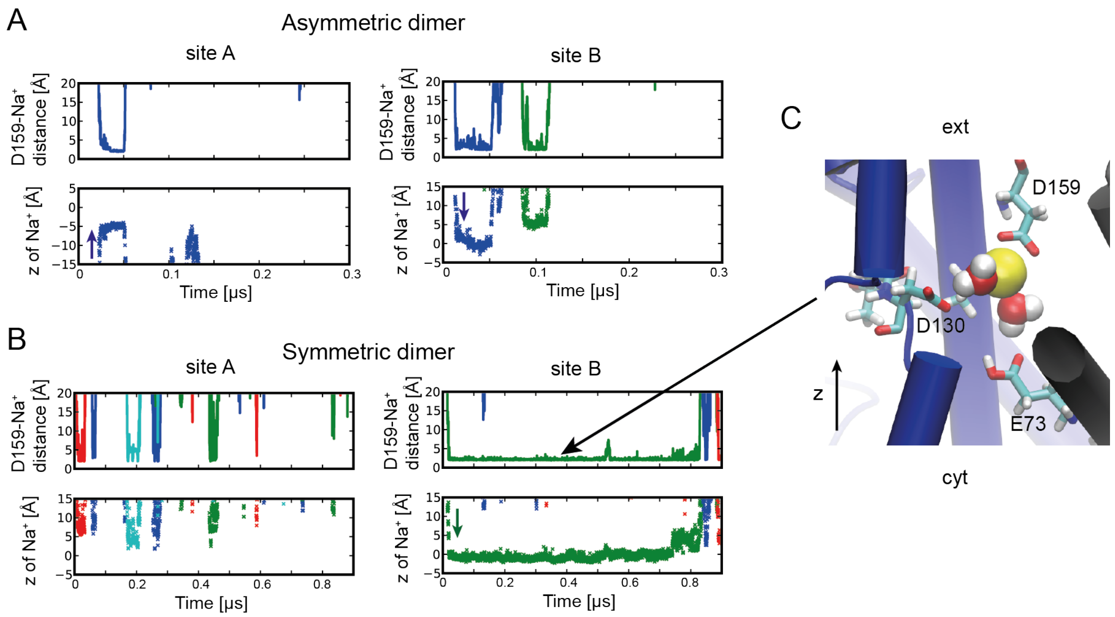

Supplementary Figure 15 | Ion-binding events in simulations with Asp159 ionized. (**A**, **B**) Distances between Na^+ and Oδ atoms of Asp159 (top), and z coordinates of the Na^+ (bottom) for site-A (left) and site-B (right). Trajectories of Na^+ ions are shown only when their distance from Asp159 is below 3 Å and remains bound for at least 4 ns. (**A**) Trajectories for the asymmetric dimer. (**B**) Trajectories for the symmetric dimer. (**C**) Snapshot of bound Na^+ (yellow sphere) in the outward-open state of the symmetric dimer trajectory.

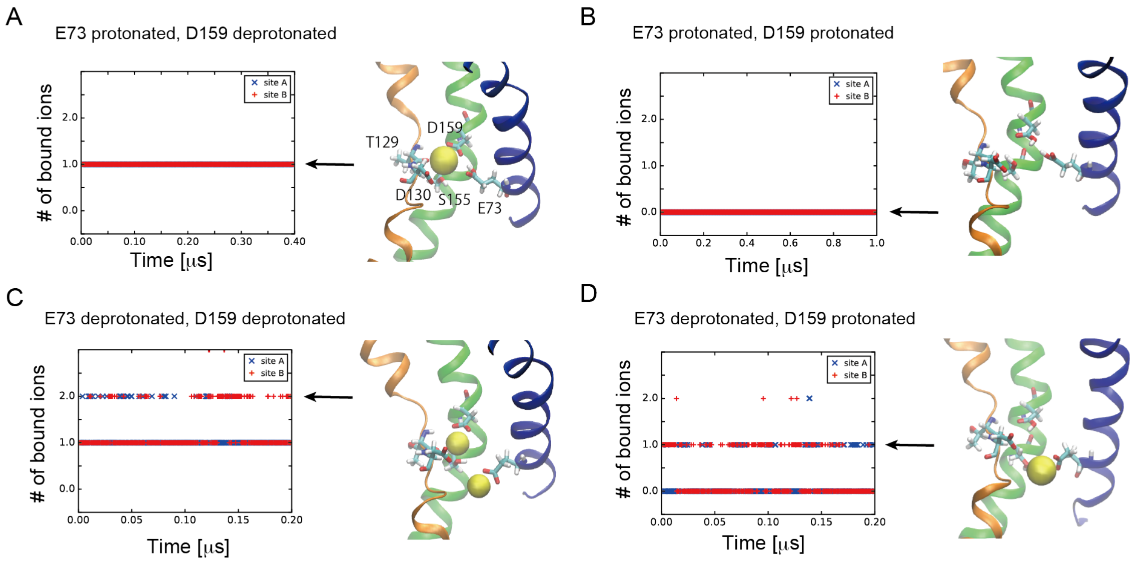

Supplementary Figure 16 | Protonation states of the ion-binding site. Ion-binding mode with different protonation states of the acidic residues in the binding site. If a sodium ion is within 3 Å distance of any atom of binding site residues (E73, T129, D130, S155 and D159), it is counted as a bound ion. (A) Simulation with E73 protonated and D159 deprotonated. The number of bound ions is plotted for the binding sites of monomer A and B in blue and red, respectively. The dominant ion-binding mode is shown with bound Na^+ as yellow spheres, the binding site residues as sticks, and the three helices holding these residues in cartoon representation. Note that this binding mode is essentially the one seen in the crystal structure. (B-D) Simulations with E73 protonated and D159 protonated (B), E73 deprotonated and D159 deprotonated (C), and E73 deprotonated and D159 protonated (D). In summary, deprotonated D159 binds Na^+ strongly, while deprotonated E73 occasionally binds and releases additional Na^+.

A

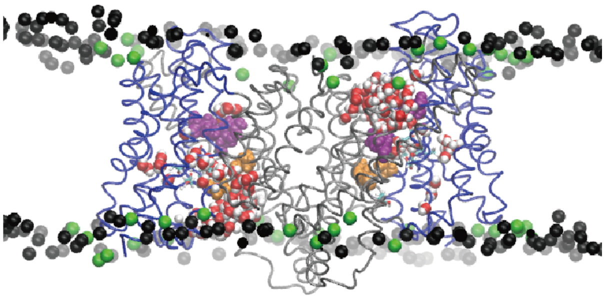

B

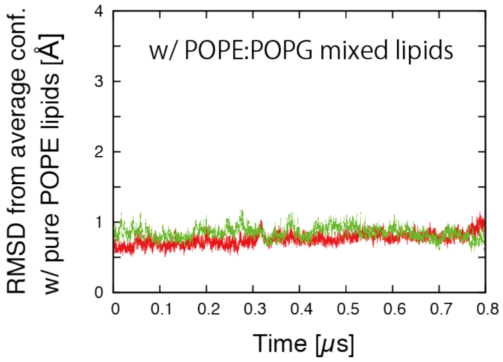

Supplementary Figure 17 | Membrane deformation and effect of lipids. (A) Lipid headgroups around the asymmetric dimer conformation defining a local membrane height profile. The last snapshot of 1 μ s equilibrium MD of the asymmetric dimer with pure POPE membrane is shown. Phosphate atoms of lipid molecules within 5 Å distance of the protein are shown in green spheres, otherwise they are shown in black spheres. (B) RMSD of monomers A (red) and B (green) in POPE:POPG membrane from the average structure in a pure POPE membrane. An equilibrium simulation in a mixed POPE:POPG lipid membrane was started from the final snapshot of the 1- μ s equilibrium MD of the asymmetric dimer in pure POPE membrane. The RMSD is calculated relative to the structure in POPE averaged over the last 0.5 μ s of MD.

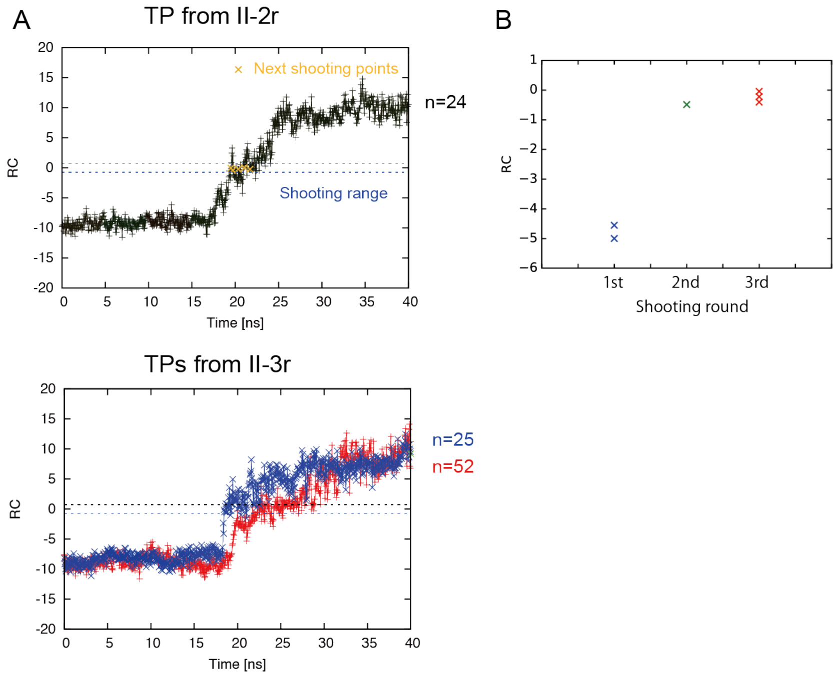

Supplementary Figure 18 | Weight factor of transition paths and successful shooting points. (A) Optimized reaction coordinate trajectories are plotted for transition paths from 2nd and 3rd rounds of transition path shootings. The boundaries of the shooting range are shown as broken lines. The numbers of points (saved at a 50-ps interval) in the shooting range are shown for each trajectory. (B) Optimized reaction coordinates for successful shooting configurations through shooting rounds following the second initial path (TMD from the outward-open to inward-open conformation). Starting from the inward-open side of the reaction coordinate in round 1, they move toward the transition-state region in round 2 (RC~0) and remain there in round 3.

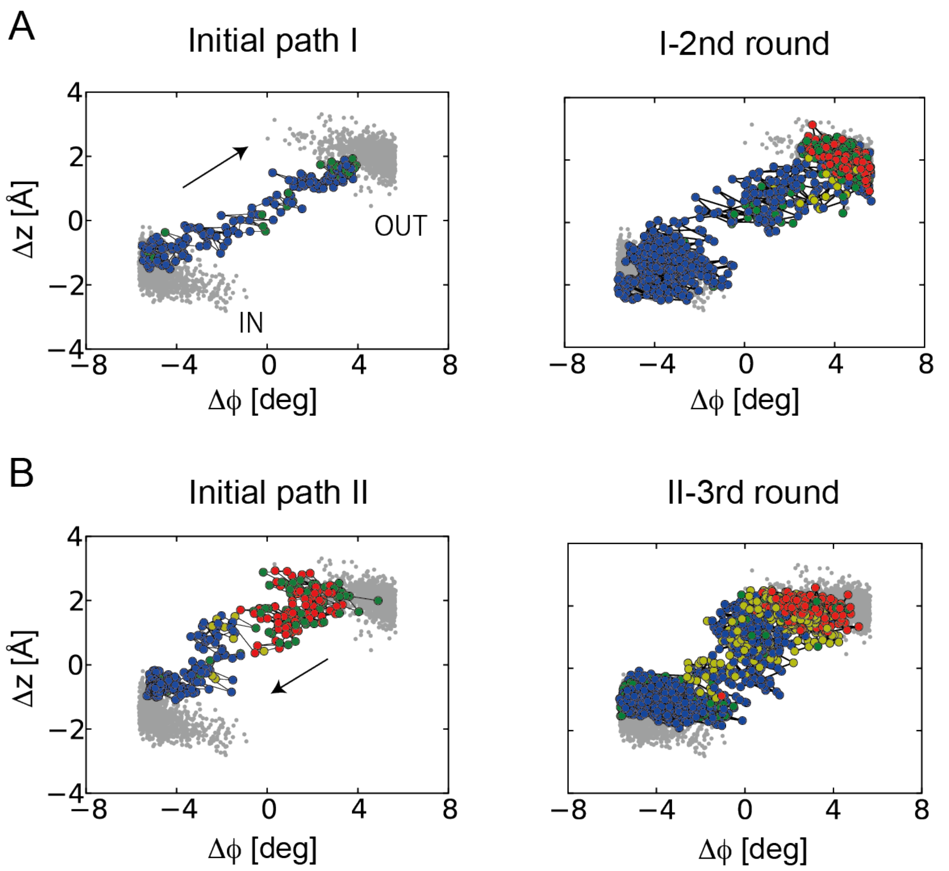

Supplementary Figure 19 | Coupling between the domain motion and water-access state of the ion-binding site in initial paths and following transition paths. (A) Initial path I from TMD simulation from the inward-open to outward-open conformation (left; TMD direction is indicated by an arrow) and following transition path (right) are projected on the $\Delta\phi$-Δz plane of the domain motion. The inward, outward, occluded and connected water-access states are indicated by blue, red, green and yellow points, respectively. (B) Initial path II from TMD simulation from the outward-open to inward-open conformation (left; TMD direction is indicated by an arrow) and following transition path (right) are projected on the $\Delta\phi$-Δz plane.

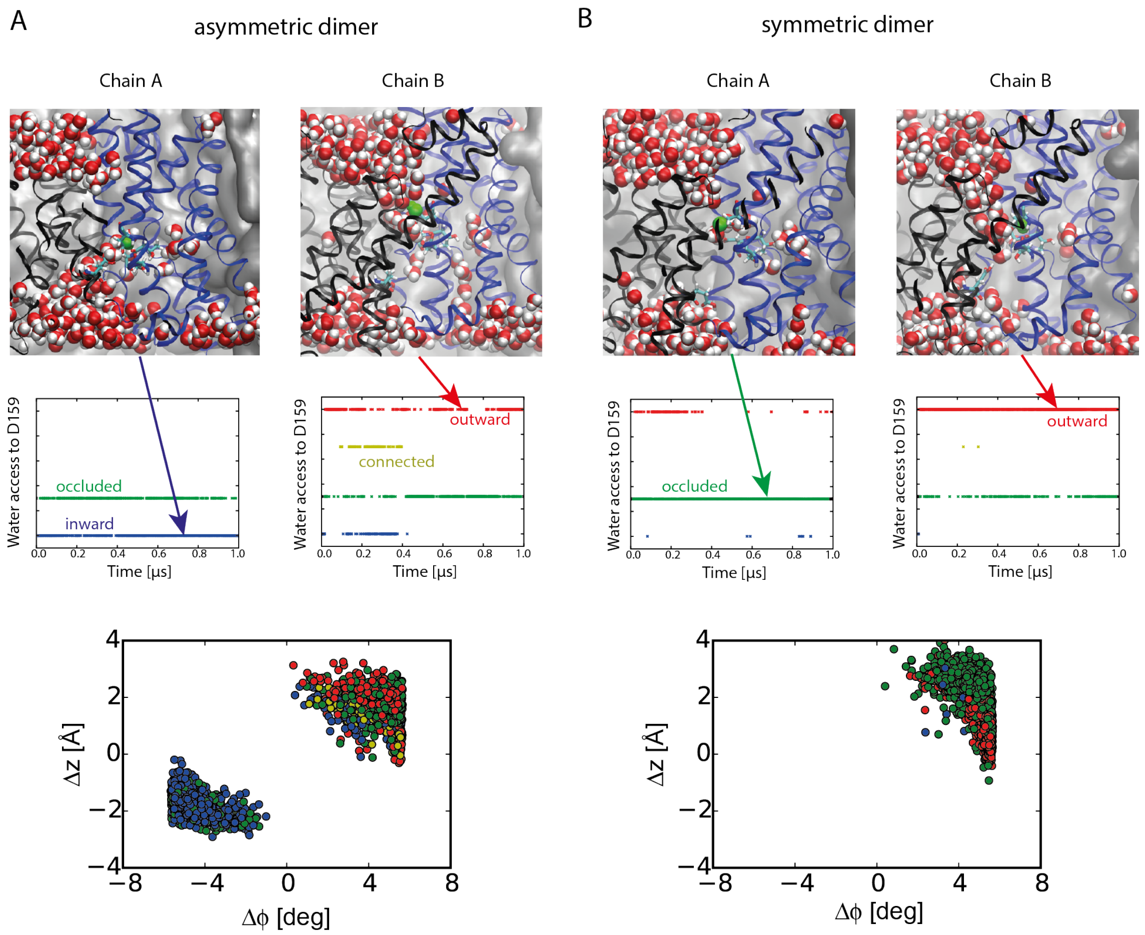

Supplementary Figure 20 | Water accessibility to the ion-binding site. Water molecules are clustered into groups by their pairwise distance, and the cluster of water molecules interacting with the carboxyl Oδ atom of D159 (green) is analysed. (**A**) Asymmetric dimer and (**B**) symmetric dimer. (Top) Typical snapshots with protein backbone as ribbons, water molecules as red (oxygen) and white spheres (hydrogen), and lipids in surface representation. Middle row: Water access state during the 1-µs trajectories, with four distinct states: inward-open (blue), occluded (green), connected (gold), and outward-open (red). Bottom row: Projection of 1-µs trajectories onto Δϕ-Δz plane reporting on transporter domain motion. Symbol colours indicate water access state.

	H^+, T=310K	H^+, T=373K	Na^+, T=310K	Na^+, T=373K		
**TS candidates**	**traj/in/out/tp**	**traj/in/out/tp**	**traj/in/out/tp**	**traj/in/out/tp**	$\Delta\phi$	Δz
			**Initial path I**			
I-1r/6ns	5/5/0/*				2.57	1.2
I-1r/7ns	5/3/0/*				2.73	1.24
I-1r/8ns	5/3/0/*				3.38	1.4
I-1r/9ns	5/1/2/*				3.03	1.7
I-1r/10ns	5/0/3/*				3.51	1.71
I-2r/A	36/22/6/1	14/13/0/0	12/8/2/2	12/4/7/2	1.05	1.04
I-2r/B	14/7/7/1	8/4/1/0	12/6/4/2	12/9/2/2	1.03	0.45
I-2r/C	8/4/0/0	8/4/1/0	12/8/0/0	12/9/1/1	1.44	0.21
I-3r/A	2/1/1/1				3.77	1.21
I-3r/B	10/2/6/1	4/3/1/1			3.17	1.48
I-4r/A	4/4/0/0	8/4/2/0			3.05	-0.1
			**Initial path II**			
II-1r/6ns	5/0/5/*				0.46	1.49
II-1r/8ns	5/0/5/*				-2.17	1.29
II-1r/9ns	5/0/0/*				-2.00	0.45
II-1r/9.2ns	5/1/0/*				-2.52	-0.17
II-1r/9.3ns	5/2/1/*				-2.28	-0.44
II-1r/9.5ns	5/3/1/*				-2.62	-0.24
II-1r/10ns	5/5/0/*				-2.70	-0.26
II-2r/A	10/0/3/0		10/0/6/0		-1.73	0.92
II-2r/B	10/5/1/1	10/1/7/0	10/7/1/1		-1.42	0.26
II-2r/C	10/0/3/0				-1.57	1.39
II-2r/D	10/0/3/0				-0.08	1.58
II-2r/E	10/0/4/0				2.22	0.99
II-3r/A	20/6/4/0	10/2/4/1			-3.02	0.50
II-3r/B	20/6/4/0	10/5/4/3			-2.40	0.92
II-3r/C	20/8/5/1	10/0/3/0			-1.98	0.29

II-3r/D	20/1/10/1	10/1/5/0			-1.19	1.17
II-3r/E	20/0/9/0	10/0/4/0			0.58	1.17

Supplementary Table 1. Transition-path shooting statistics. Column 1 labels transition-state candidates, defined as configurations from which trajectories were initiated, according to the four/three rounds of the iterative transition-path shooting following initial paths I/II (I-1r to I-4r / II-1r to II-3r). The configurations of the first round are labelled in addition by the time point at which they were sampled in the TMD trajectory. In column 2-5, the number of total trajectory shots (traj), trajectories that end up in the inward-open state (in), outward-open state (out), and successful transition paths (tp) are listed, respectively, for states with H^+ and Na^+ bound, and for normal and elevated temperatures. The star indicates that in the first round, trajectory segments were not initiated with sign-inverted initial velocities and could thus not be stitched together to form true transition paths. In column 6 and 7, the rotational angles (degrees) and *z*-translations (Å) of the six-helix-bundle domain are listed.

	H^+, T=310K		H^+, T=373K		Na^+, T=310K		Na^+, T=373K	
	**# traj**	**duration**	**# traj**	**duration**	**# traj**	**duration**	**# traj**	**duration**
eq in	1	1.0	1	0.4	1	0.4	1	0.3
eq a-out	1	1.0			1	0.5		
eq s-out	1	1.0			1	1.0		
Initial path I								
1r TPS	25	0.01						
2r TPS	92/22	0.04/0.02	30	0.02	36	0.02	36	0.02
3r TPS	24	0.02	4	0.02	24	0.02	10	0.02
4r TPS	16	0.02	8	0.02				
Initial path II								
1r TPS	35	0.02						
2r TPS	50	0.02	10	0.02	20	0.02		
3r TPS	100	0.02	50	0.02				
Total	11.87 μs		2.44 μs		3.5 μs		1.22 μs	

Supplementary Table 2. Computational effort. The equilibrium and transition-path shooting trajectories are listed as “eq” and “*n*r TPS”, respectively, where *n* represents *n*-th round of transition-path shootings. “in”, “a-out,” and “s-out” refer to inward-open, asymmetric inward/outward-open, and symmetric outward-open states, respectively. All times are in units of microseconds. The total simulation time was ~19 μs.

Name	PDB	Conserved ASP	Gate
**PaNhaP**	4CZA	D159	I163, Y255
			V365, L369
**MjNhaP1**	4CZB	D161	I165, Y248
			V350, L354
**NhaA**	1ZCD	D163,	I168, T227
		D164	F344, I345
**NapA**	5BZ2	D156,	L161,I238,V239
		D157	I337

Supplementary Table 3. Gate residues predicted from structural alignment of inward-open conformations. For each structure, the main gate residues are in the upper line, while complementing residues are in the lower line.
