# Supplementary material for: Mechanism of the electroneutral sodium/proton antiporter PaNhaP from transition-path shooting
Source: Nat Commun. 2019 Apr 15;10:1742. doi: 10.1038/s41467-019-09739-0 (PMC6465308; doi:10.1038/s41467-019-09739-0)
Supplement: Supplementary file 3 — Description of Additional Supplementary Files [file 41467_2019_9739_MOESM3_ESM.pdf]

Description of Supplementary Files

File Name: Supplementary Movie 1

Description: Representative transition paths are shown. In the first half, the transition path with Na+ bound (yellow sphere) from the inward to outward-open conformation is shown. In the last half, the transition path with H+ bound (green sphere) from the outward to inward-open conformation is shown.

File Name: Supplementary Data 1

Description: The asymmetric dimer conformation of PaNhaP obtained by the simulation is provided in PDB format.
